# Listeria spondylodiscitis: an uncommon etiology of a common condition; a case report

**DOI:** 10.1186/s12879-020-05286-y

**Published:** 2020-07-31

**Authors:** Rand Al Ohaly, Nischal Ranganath, Medina G. Saffie, Anjali Shroff

**Affiliations:** 1 Department of Medicine, McMaster University, Hamilton, ON Canada; 2 Joseph Brant Hospital, Burlington, ON Canada

**Keywords:** Listeria, Spondylodiscitis, Bone & joint infection, Listeriosis, Case report

## Abstract

**Background:**

Listeriosis is a severe food-borne infection caused by the Gram-positive rod, *Listeria monocytogenes.* Despite the low incidence (3–8 cases per million), Listeriosis has a case fatality rate of 20–30% as it occurs predominantly in immunocompromised individuals at extremes of age, diabetics and pregnant women. Listeriosis classically presents as a febrile gastroenteritis, isolated bacteremia, meningitis, or maternal-fetal infections. Focal bone and joint infection are rare and primarily involve orthopedic implant devices. Here, we present the first case of Listeria-associated spondylodiscitis.

**Case presentation:**

A 79-year-old male presents with acute-on-chronic back pain in the absence of risk factors or exposures, aside from age. On radiological imaging, spondylodiscitis of L3-L4 was diagnosed. Subsequently, a CT-guided biopsy was performed to aid in confirming microbiological aetiology. *Listeria monocytogenes* was grown in culture and patient received appropriate antibacterial therapy.

**Conclusion:**

The case highlights the utility of image-guided tissue sampling in aiding diagnosis and management in patients with vertebral osteomyelitis. It also encourages consideration of uncommon organisms such as *Listeria* as an etiology of vertebral osteomyelitis, even in the absence of prosthetic implants.

## Introduction

*L. monocytogenes* is a gram-positive, facultative, intracellular bacillus responsible for the febrile gastrointestinal illness, Listeriosis, acquired from contaminated food that typically occurs sporadically or in outbreaks [1]. There are several serotypes of Listeria distinguished based on the cell-surface O and H antigens, with the three serotypes 1/2c, 1/2b, and 4b accounting for greater than 95% of human illnesses [2]. The organism possesses several features including an intrinsic resistance to high acidity and salinity, as well as the capacity to grow at low temperatures that support it’s efficacy in foodborne transmission [3]. Following ingestion, systemic infection can be established through tissue penetration across the gastrointestinal tract or hematogenous spread to sterile sites including the central nervous system (CNS), heart, liver, spleen, and placenta [3]. Consistent with this, *L. monocytogenes* associated bacteremia, meningoencephalitis, infective endocarditis, and endovascular graft infections have been previously reported [4–7]. Interestingly, however, this organism rarely causes bone and joint infections, and if so, occurs typically in the context of prosthetic material providing a site for bacterial seeding. Herein, we present the first case of Spondylodiscitis in an individual void of known risk factors.

## Case presentation

A 79-year-old gentleman presented with a 3-week history of acute, progressively worsening right-sided back pain radiating to the right groin and anterior thigh. He was admitted due to reduced mobility secondary to pain and an inability to safely function independently at home. A risk assessment for red flag symptoms including spinal cord or cauda equina compression, progressive neurological deficits, new urinary retention, faecal incontinence, and constitutional symptoms were absent. He was in his usual state of health prior to the onset of these symptoms. He denied any history of fevers, rigors, gastrointestinal symptoms, inciting trauma, or recent dental or surgical procedures. Social history included a 30-pack-year smoking history, minimal alcohol use, and denied intravenous substance use. His past medical history included hypertension, dyslipidaemia, gout and reflux. His past surgical history involved bilateral hernia repair with mesh, bilateral subclavian to carotid artery bypass and repair of an endovascular infrarenal abdominal aortic aneurysm, which required embolization for a leak at the left internal iliac artery 4 years later. Home medications included Clopidogrel, Hydrochlorothiazide, Perindopril, Allopurinol, Pantoprazole, Rosuvastatin, and Tamsulosin. There was no history of immunosuppression or use of immune modulating medications, particularly glucocorticoids. He denied any recent travel, animal exposures, consumption of processed meats, cheeses or other dairy products.

The patient was afebrile and hemodynamically stable at presentation and throughout his hospital stay. Physical examination of the cardiorespiratory, abdominal, and neurological systems was within normal limits. A focused examination revealed no tenderness on spinal palpation, a negative straight leg raise test, no pain on leg roll, and a normal gait. Relevant laboratory results included lymphopenia (0.7 × 10^9^/L), anaemia (haemoglobin 101 g/L), LDH of 202 U/L, and CRP 34.9 mg/L, which later increased to 87.9 mg/L. Multiple sets of blood cultures were repeatedly negative. During his admission, he had an episode of self-resolving diarrhea. Interestingly, despite no prior antibiotic exposure, a stool sample was positive for *C. difficile* toxin by PCR. This, however, was more representative of a colonisation rather than a true infection.

**Fig. 1:**
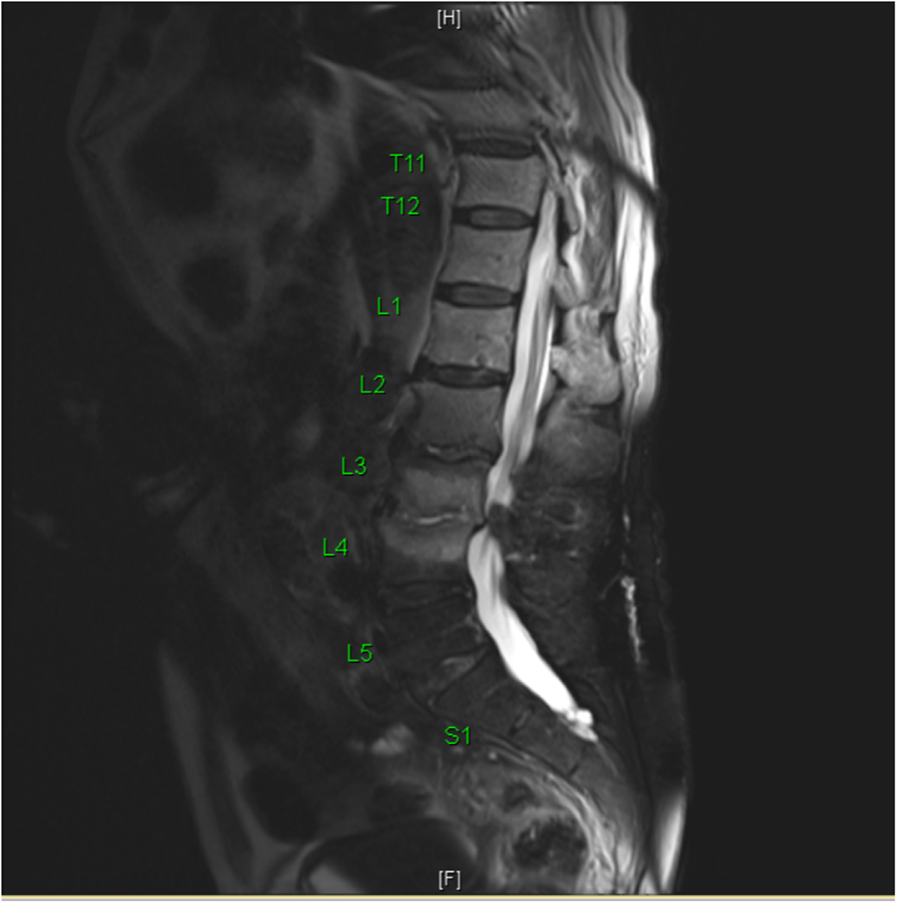
Sagittal T2 Fat Sat: high signal intensity within the L3-L4 disc space involving the inferior endplate of L3 and superior end plate of L4 with end plate irregularity

**Fig. 2:**
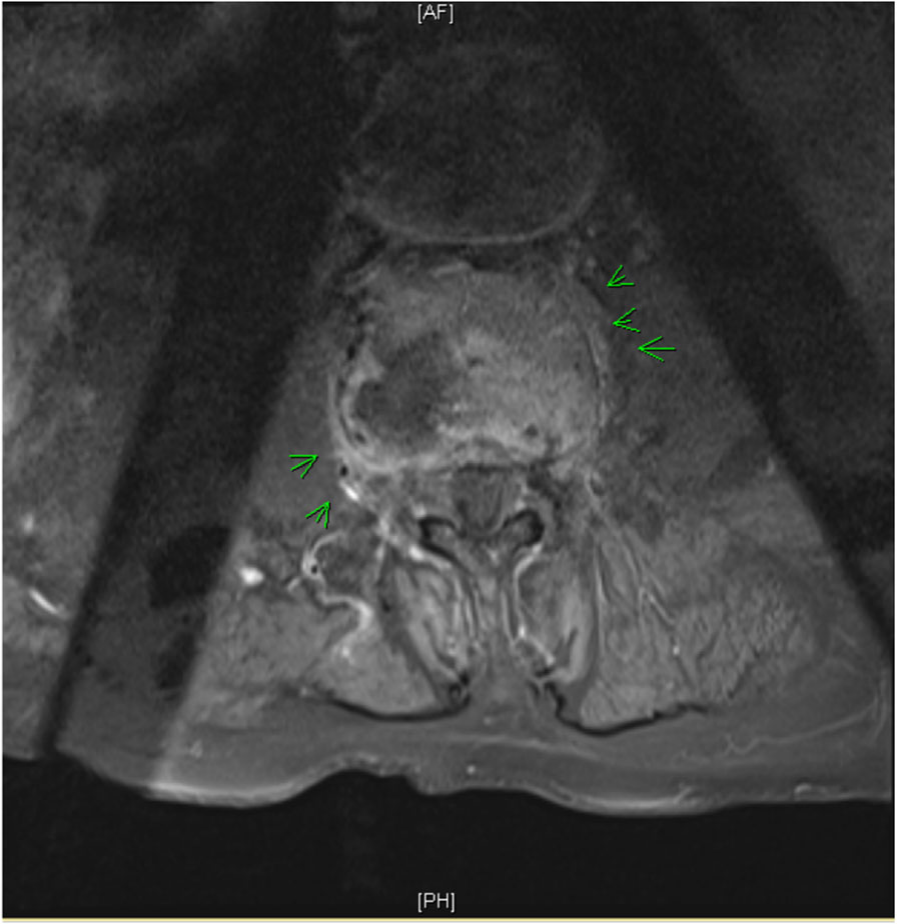
Axial T1 post-gadolinium: (no pre-GAD) images. Small amount of enhancing paravertebral soft tissue. No evidence to suggest paravertebral or epidural abscess formation

Radiographs of the hip and pelvis showed multi-level degenerative disc disease without evidence of fracture. CT Chest/Abdomen/Pelvis with endovascular protocol was negative for endoleak or dissection, but incidentally demonstrated interval L3-L4 discitis with destruction of associated endplates. Transthoracic echocardiogram effectively ruled out the presence of valve disease or vegetations. An MRI with gadolinium was subsequently performed and confirmed the diagnosis of L3-L4 discitis and osteomyelitis (Figs. 1, 2). Given the clinical stability and a lack of clear causative pathogen on blood culture, antibiotic administration was delayed to accommodate a fluoroscopy-guided percutaneous aspiration and biopsy. Fluid aspirate returned positive for *Listeria monocytogenes* in broth culture. Fungal and mycobacterial cultures remained negative. *L. monocytogenes* was thus determined to be the aetiology and the patient was started on a 6-week course of Ampicillin, with rapid clinical improvement and normalization of CRP on serial assessment. The patient was subsequently seen in follow-up with both the Infectious Disease and Vascular Surgery services at 2- and 6-month time points with ongoing clinical recovery and no recurrence of disease.

## Discussion and conclusions

Despite its rarity (between 3 to 8 cases per million worldwide), the average case-fatality rate of invasive Listeriosis is up to 20–30%, even with appropriate antimicrobial therapy [8, 9]. This is largely attributable to a predilection for immunocompromised individuals with extremes of age (neonates and elderly age ≥ 65 years), diabetics and pregnant women. Additional high-risk populations include those with hematologic malignancy, end-stage renal disease, AIDS, organ transplantation, iron-overload states, alcoholism and other chronic liver diseases [3, 8]. In particular, the risk of invasive Listeriosis is noted with the use of TNF-α inhibitors as it suppresses T-cell mediated immunity; the primary defence against this organism [10–12].

Listeriosis classically presents as febrile gastroenteritis, isolated bacteremia, meningitis, encephalitis, or maternal-fetal infections [2, 8]. Localized CNS involvement often presents as subcortical brain or intramedullary spinal abscesses [13–16]. Interestingly, bone and joint infection secondary to *Listeria* is uncommon and primarily involves orthopedic implant devices including prosthetic joints [11]. In the French case series of *Listeria*-associated bone and joint infections, 84% of infections involved prosthetic material, with a minority of cases involving native joint infections [11]. To the best of our knowledge, there have only been 2 prior cases of *Listeria*-associated vertebral osteomyelitis (OM) occurring in the context of risk factors including spine surgery and immunosuppression, as well as 1 case of *Listeria* bacteremia with spondylodiscitis in the absence of clear risk factors [13, 14, 17].

Here we present the first case of Listeria-associated spondylodiscitis in a 79-year-old male in the absence of significant risk factors, bacteremia, comorbidities (including prostheses), or exposures. The patient’s advanced age is the only shared risk factor with the previously published case reports. Given the non-specific presentation of back pain and absence of infectious prodrome, the finding of vertebral spondylodiscitis was made incidentally. Further investigation of the spondylodiscitis yielded repeated negative blood cultures; thus, necessitating a CT-guided biopsy and aspiration for diagnosis.

### Diagnostic yield of biopsy

The determination of a microbiologic diagnosis, as in this case, can be challenging in the absence of obvious exposures, negative blood cultures, and serology. In these circumstances, fluoroscopy-guided diagnostic aspiration biopsy is valuable in obtaining samples for microbiologic testing and in providing diagnostic clarity between infectious, malignant or degenerative processes [18]. CT-guided biopsy has variable sensitivity in determining infectious etiology ranging from 30.4 to 74% [18–20], with a recent metanalysis demonstrating 52.2% (95% CI, 45.8–58.5%) sensitivity and 99.9% (95% CI, 94.5–100%) specificity [21]. This strategy therefore represents an excellent modality to obtain a microbiologic diagnosis; but the absence of a positive culture does not necessarily rule out infection. From a patient outcome standpoint, CT-guided aspiration for culture also prevents the need for open surgical intervention and biopsy in 50–60% of cases [18] and can facilitate targeted antibiotic therapy [22].

### Timing of antibiotics in relation to diagnostic sampling

As in this case, empiric therapy should be delayed, if the patient is haemodynamically stable with no neurological compromise, in order to obtain tissue samples for culture as per the IDSA guidelines for native vertebral osteomyelitis management [13, 17]. The postponement of antibiotic therapy does improve microbiological yield and can be deferred in the absence of life-threatening conditions or spinal cord compromise [23]. However, initiation of antibiotics does not preclude undertaking a biopsy, as a recent metanalysis demonstrated a non-significant difference in biopsy yield of 32% vs 43% in those with and without prior antibiotic exposure [24]. In cases where antibiotic therapy has already been started, it was demonstrated that interrupting and withholding antibiotics for approximately 14 days pre-CT guided tissue biopsy had a better yield (76.9%) when compared to holding for only 3 days pre-biopsy (41.2%) [25]. This can vary, however, depending on the pharmacokinetics, dose and duration, and bone penetration of the selected empiric antibiotic. Nevertheless, a short duration of empiric antibiotic exposure does not negatively impact pathogen recovery and is therefore not a contraindication for vertebral biopsy [26]. If image-guided sampling of tissue is not feasible or available, or signs of clinical instability or focal neurological involvement is identified, appropriate empiric therapy and timely consultation of the Infectious Diseases and Spinal Surgery services is warranted [18].

### Conclusion

In summary, this case report presents the first case of *Listeria*-associated spondylodiscitis in the absence of a clear exposure, wherein the only risk factor was age. Consideration for Listeria-associated osteomyelitis should be a part of the differential diagnosis, even in the absence of prosthetic material. This is particularly important in those with appropriate epidemiologic risk factors, but should also be considered in those living in areas with relatively low incidences of *Listeria* infection as in this case. Lastly, the present case strongly supports the utility of tissue sampling in all cases of suspected OM, that have no ongoing neurological or hemodynamic compromise, in order to achieve microbiological etiology. This can facilitate the use of the narrowest spectrum and most effective antibiotic appropriate for optimal patient care.

## Data Availability

Not applicable.

## Abbreviations

CNS: Central nervous system
OM: Osteomyelitis
